# Familial adult-onset neuronal intranuclear inclusion disease: A case report and literature review

**DOI:** 10.1097/MD.0000000000040279

**Published:** 2024-11-01

**Authors:** Lijun Wei, Jiaqi Wang, Changming Xu, Tengchao Yang, Yun Tian, Lu Shen

**Affiliations:** a Department of Neurology, Huludao City Center Hospital, Huludao, Liaoning, China; b Baotou Medical College, Inner Mongolia University of Science and Technology, Baotou, China; c Jinzhou Medical University, Jinzhou, Liaoning, China; d Department of Neurology, Xiangya Hospital, Central South University, Changsha, Hunan, China.

**Keywords:** episodic encephalopathy, NIID, *NOTCH2NLC* gene, p62 antibody-positive intranuclear inclusion bodies

## Abstract

**Rationale:**

Neuronal intranuclear inclusion disease (NIID) is a rare neurodegenerative disorder with highly variable clinical manifestations, making diagnosis challenging. Recent advancements in genetic and pathological testing, such as the identification of GGC repeat expansions in the *NOTCH2NLC* gene, have improved diagnostic accuracy, but familial cases remain underreported.

**Patient concerns:**

This report details 3 cases of familial adult-onset NIID in 2 sisters and 1 brother. The older sister experienced episodic encephalopathy and autonomic dysfunction for over 40 years, while the younger sister presented similar symptoms 5 years ago. The brother also developed episodic encephalopathy 5 years ago. Brain diffusion-weighted imaging (DWI) for all 3 patients revealed hyperintensity at the corticomedullary junction and corpus callosum. Skin biopsies from the older sister and brother confirmed the presence of p62 antibody-positive intranuclear inclusion bodies in sweat gland cells and fibroblasts. Genetic testing showed 146 and 133 GGC repeats in the *NOTCH2NLC* gene in the older sister and brother, respectively.

**Diagnoses:**

All 3 patients were diagnosed with NIID based on clinical, radiological, and genetic findings.

**Interventions:**

The patients received hormonal therapy, circulation-enhancing treatments, and rehydration therapy during acute episodes.

**Outcomes:**

All 3 patients showed significant improvement in symptoms following treatment, with a return to baseline function after hospital discharge.

**Lessons:**

Proper management of NIID includes prompt recognition of symptoms, adequate rest, and avoidance of triggers such as fatigue, infections, and stress, which may reduce the frequency of episodic attacks. Early diagnosis and targeted symptomatic treatment are essential for improving patient outcomes.

## 1. Introduction

Neuronal intranuclear inclusion disease (NIID) is a rare neurodegenerative disorder with highly heterogeneous clinical manifestations.^[1,2]^ Depending on the initial symptoms and main clinical features, NIID can be classified into four subtypes: dementia type, movement disorder type, muscle weakness type, and paroxysmal symptom type.^[3]^ Based on family history, NIID is further divided into sporadic and familial types, and depending on the age of onset, it can be categorized into childhood, juvenile, and adult types.^[4]^ In adult-onset cases, NIID can mimic other neurological diseases such as Alzheimer’s disease, Parkinson’s disease, and multiple system atrophy, making early diagnosis particularly challenging.

In recent years, an increasing number of NIID cases have been diagnosed through skin biopsy,^[5]^ demonstrating the growing recognition of the disease. However, familial NIID cases remain rare, and few reports have been published to date. Here, we present 3 rare familial adult-onset NIID cases from a Chinese family, diagnosed through a combination of clinical evaluation, neuroimaging, genetic testing, and skin biopsy. By documenting these rare familial cases, this report contributes to the growing body of knowledge regarding this rare disorder, helping clinicians better recognize and diagnose NIID in its early stages, particularly in familial contexts.

## 2. Case presentation

### 2.1. Patient 1

The first patient is a 77-year-old female with a history of episodic neurological symptoms spanning more than 40 years. She first presented with recurrent episodes of dizziness, nausea, and vomiting. Fifteen years ago, she experienced her first episode of consciousness disturbance, and these episodes have recurred every 4 to 5 years, typically resolving within 1 to 2 days without lasting deficits. Eight years ago, she had a more severe episode with aphasia, left limb weakness, and fever, which again resolved within 2 days. Recently, she developed difficulty in urination, requiring assistance, which led to recurrent urinary tract infections. In June 2023, she experienced another episode of unsteady gait and exacerbation of her prior symptoms.

On physical examination, the patient exhibited muscle atrophy in both lower limbs, with muscle strength graded as IV. Neurological examination showed dysmetria in the finger-nose test and instability in the heel-knee-tibia test. She also struggled to stand with her eyes closed. Her cognitive assessments were relatively preserved, with Montreal Cognitive Assessment and Mini-Mental State Examination scores of 26 and 27, respectively.

**Figure 1.**
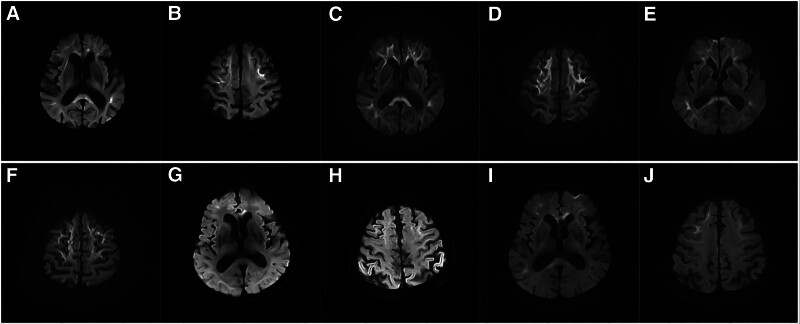
Brain MRI findings of the 3 patients. (A, B) show findings for Patient 1. Brain DWI in May 2019 shows hyperintensity in the corticomedullary junction of the bilateral frontoparietal, temporal, and occipital lobes and genu and splenium of the corpus callosum. (C, D) Brain DWI of the same patient in June 2023 shows hyperintensity in the corticomedullary junction of the bilateral frontoparietal, temporal, and occipital lobes and genu and splenium of the corpus callosum. Over the 4-year period from 2019 to 2023, the lesions had expanded to the posterior part of the brain, and the white matter lesions were slightly worse than those observed before. (E, F) show findings for Patient 2. Brain DWI in October 2023 shows hyperintensity in the corticomedullary junction of the bilateral frontoparietal, temporal, and occipital lobes and genu and splenium of the corpus callosum. (G–J) show findings for Patient 3 from 2020 to 2023; (G–H) represent findings in April 2020; (I–J) represent findings in May 2023. DWI hyperintensity was noted in the corticomedullary junction of the bilateral frontoparietal, temporal and occipital lobes and genu and splenium of the corpus callosum. The lesion expanded slightly to the posterior part of the brain over the 3-year period from 2020 to 2023. DWI = diffusion-weighted imaging, MRI = magnetic resonance imaging.

**Figure 2.**
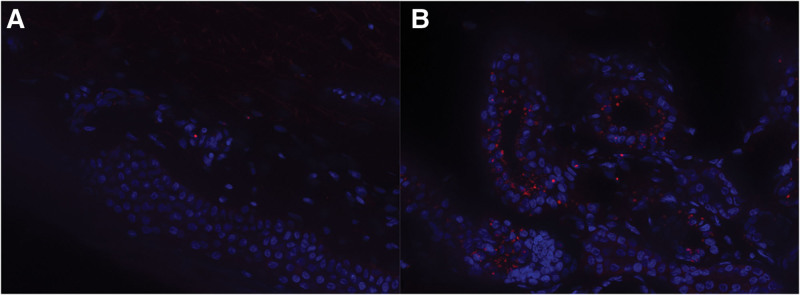
Results of skin biopsy examination. (A) In Patient 1, p62 antibody-positive inclusion bodies (IBs) were detected in the nuclei of skin fibroblasts. (B) In Patient 3, p62 antibody-positive IBs were found in the nuclei of sweat gland ductal epithelial cells.

**Figure 3.**
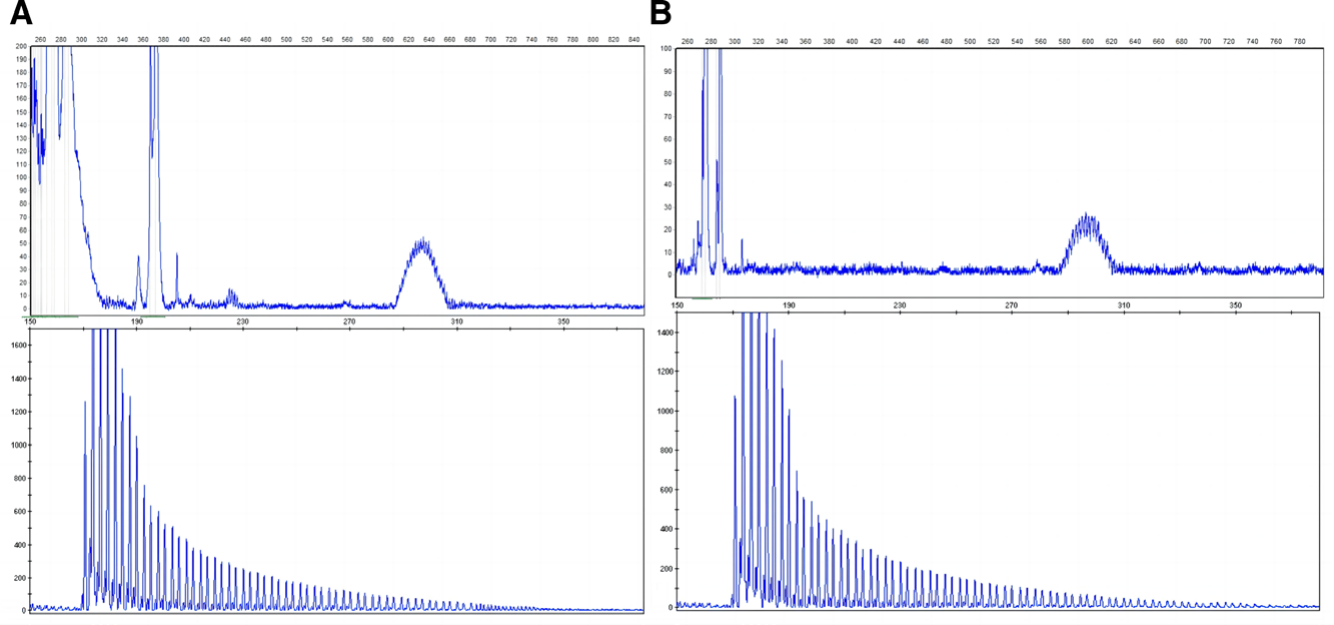
Results of genetic testing. (A) shows 146 repeats GGC repeat amplification in the *NOTCH2NLC* gene in Patient 1. (B) shows 133 repeats GGC repeat amplification in the *NOTCH2NLC* gene in Patient 3.

Serial diffusion-weighted imaging (DWI) of the brain, performed in 2019 and 2023, demonstrated hyperintensity at the corticomedullary junction, bilaterally in the frontoparietal, temporal, and occipital lobes, as well as in the genu and splenium of the corpus callosum. These lesions progressed over time, with worsening white matter involvement (Fig. 1A–D). Skin biopsy showed p62 antibody-positive intranuclear inclusion bodies (IBs) in skin fibroblasts (Fig. 2A), and genetic testing revealed 146 GGC repeats in the *NOTCH2NLC* gene (Fig. 3A). Based on these findings, the patient was diagnosed with neuronal intranuclear inclusion disease (NIID). During hospitalization, she was treated with therapies aimed at improving blood circulation, rehydration, and anti-inflammatory agents. Upon discharge in June 2023, her symptoms had returned to baseline, and her extrapyramidal symptoms were notably improved.

### 2.2. Patient 2

The second patient, a 71-year-old female, was admitted in October 2023 with a 5-year history of progressive motor slowness and unsteady gait. Over this period, she experienced multiple falls, episodes of confusion, and urinary incontinence. Two months prior to her admission, she developed slurred speech and reduced mobility in her right limb. Her family also noted occasional episodes of disorientation, including 1 incident where she could not find her way home.

On examination, she had dysarthria and rigidity in her right limbs. Brain DWI demonstrated hyperintensity at the corticomedullary junction and corpus callosum, similar to findings in her older sister (Fig. 1E,F). During hospitalization, she received symptomatic treatment aimed at improving circulation and rehydration. At her 1-month follow-up, her condition had returned to baseline, although she still experienced slight instability when walking.

### 2.3. Patient 3

The third patient, a 63-year-old male, presented 5 years ago with episodic severe headaches lasting 1 to 2 days. Three years ago, he developed headaches accompanied by nausea, lethargy, generalized weakness, and cognitive decline, which recurred intermittently along with high fever. Despite these recurring episodes, cerebrospinal fluid (CSF) examination was normal, with normal opening pressure and unremarkable cell and protein counts. Over time, his cognitive symptoms progressed, and he experienced multiple episodes of encephalopathy.

Brain DWI performed over the course of several years showed hyperintensity at the corticomedullary junction and the splenium of the corpus callosum (Fig. 1G,H). These lesions expanded posteriorly over time (Fig. 1I,J). A skin biopsy revealed p62 antibody-positive IBs in the nuclei of sweat gland epithelial cells (Fig. 2B), and genetic testing identified 133 GGC repeats in the *NOTCH2NLC* gene (Fig. 3B). Based on these findings, the patient was diagnosed with NIID. He was hospitalized 3 times in 2023 and received hormonal therapy, enhanced blood circulation treatment, and rehydration. At his most recent follow-up, conducted 1 month after discharge, his symptoms had fully recovered to baseline.

## 3. Discussion

This case report presents 3 familial adult-onset NIID cases, with detailed descriptions of their clinical manifestations, supported by findings from neuroimaging, genetic testing, and pathology. The comprehensive approach used in this report provides valuable insights for the early diagnosis and clinical management of NIID.

Episodic encephalopathy emerged as a critical diagnostic feature in all 3 patients. The episodes included consciousness disturbances, dizziness, nausea, and headaches, which are consistent with previously reported clinical presentations of NIID.^[6]^ Various studies have reported the incidence of episodic encephalopathy in NIID cases to range between 18% and 64.5%,^[1,7–9]^ but the higher frequency of such episodes observed in our patients suggests that episodic encephalopathy may be underreported. Further studies are needed to clarify whether diagnostic bias contributes to these differences.^[10]^ Additionally, autonomic nervous system dysfunction, particularly dysuria and urinary incontinence, was observed in 2 of the patients (Patients 1 and 2), reinforcing the importance of considering autonomic symptoms in the diagnostic process for NIID.^[6]^ These findings are consistent with previous literature, emphasizing the combined role of episodic encephalopathy and autonomic dysfunction in enhancing diagnostic accuracy.^[6]^

Brain diffusion-weighted imaging (DWI) is a crucial diagnostic tool for NIID. All 3 patients in this report demonstrated typical DWI hyperintensity at the corticomedullary junction and in the corpus callosum, which are well-established radiological hallmarks of NIID.^[1,11]^ These findings were consistent with previously reported cases, where DWI hyperintensity has been identified as a key diagnostic feature, particularly in Chinese studies.^[1,11]^ However, familial adult-onset NIID cases have shown lower frequencies of DWI hyperintensity, with some studies reporting an incidence of only 37.5%.^[7]^ The fact that all 3 of our patients displayed this characteristic feature suggests that DWI may be more frequently present in familial cases than previously documented. Additionally, the progressive posterior expansion of lesions over time in Patient 1 and Patient 3 aligns with the known course of NIID as described in the literature.^[11,12]^

In 2019, studies from both China and Japan identified GGC repeat expansions in the *NOTCH2NLC* gene as a key genetic marker for NIID. This discovery has significantly improved the accuracy of diagnosing NIID in both familial and sporadic cases.^[7–9,13]^ In this report, genetic testing revealed that Patient 1 had 146 GGC repeats, while Patient 3 had 133 repeats. The number of GGC repeats in these patients falls within the 100 to 200 range, which is commonly associated with clinical manifestations such as parkinsonism and myasthenia.^[7,14]^ Despite the number of repeats, neither patient exhibited signs of dementia, a finding that differs from the literature, which associates larger repeat expansions with more severe neurological phenotypes. This discrepancy suggests that further research is needed to explore the variability in the clinical presentation of NIID, as the correlation between GGC repeat length and phenotypic severity remains unclear.

Pathological examination, particularly through skin biopsy, plays a vital role in diagnosing NIID. In 2011, Sone et al^[15]^ identified p62-positive intranuclear inclusion bodies (IBs) in the nuclei of ductal epithelial cells, fibroblasts, and adipocytes in the skin, highlighting the diagnostic value of noninvasive skin biopsies. In our study, Patient 1 and Patient 3 underwent skin biopsies that confirmed the presence of p62-positive IBs in sweat gland cells and fibroblasts, which corroborates the diagnosis of NIID. These findings further validate the use of skin biopsy as a reliable, less invasive alternative to brain biopsy in diagnosing NIID, particularly in familial cases. The identification of these intranuclear inclusions is a hallmark feature of NIID and provides definitive pathological evidence to support the clinical and radiological findings. The combination of p62-positive IBs with the characteristic DWI hyperintensity and genetic markers reinforces the multi-modal diagnostic approach to NIID.

In this report, we described the clinical manifestations, neuroimaging features, genetic testing, and skin biopsy results of 3 familial adult-onset NIID patients. These cases demonstrate the diagnostic difficulties of NIID due to its broad heterogeneity. Key diagnostic indicators included episodic encephalopathy and autonomic dysfunction, with characteristic DWI hyperintensity at the corticomedullary junction providing a crucial radiological clue. We recommend considering skin biopsy and genetic testing when these clinical and imaging features are present. All 3 patients had pre-episode triggers, such as fatigue or cold exposure, and high-dose intravenous corticosteroid therapy administered early in the disease course effectively halted symptom progression and led to rapid improvement. While no definitive treatment for NIID exists, avoiding stressors and infections may reduce the frequency of episodes, and corticosteroid treatment during episodes has shown promise in rapidly improving consciousness and neurological symptoms. However, due to the limited number of cases and lack of long-term data, the efficacy of corticosteroids over the long term remains uncertain. This report provides new insights into the genetic predisposition and clinical variability of NIID, aiding in the early recognition and diagnosis of this rare condition.

This study has several limitations that should be acknowledged. First, the sample size is small, consisting of only 3 familial cases, which limits the generalizability of the findings to the broader population of NIID patients. While these cases provide valuable insights into the familial form of NIID, larger studies are needed to confirm these findings and explore potential variations in clinical presentation and genetic characteristics. Second, the follow-up period for the patients was relatively short, particularly for monitoring long-term disease progression and the efficacy of symptomatic treatments.

## 4. Conclusions

In conclusion, this case report of 3 familial adult-onset NIID patients highlights the importance of a multi-modal diagnostic approach, incorporating clinical presentations, neuroimaging, genetic testing, and pathological findings. The presence of episodic encephalopathy, autonomic dysfunction, DWI hyperintensity at the corticomedullary junction, and p62-positive intranuclear inclusion bodies provides a strong foundation for diagnosing NIID in adult-onset cases. Early and accurate diagnosis is crucial for improving patient outcomes, and further studies are needed to better understand the natural history of familial NIID and the potential for personalized treatment strategies.

## Author contributions

**Conceptualization:** Yun Tian.

**Data curation:** Lu Shen.

**Formal analysis:** Lijun Wei, Jiaqi Wang, Changming Xu, Tengchao Yang, Yun Tian.

**Investigation:** Lu Shen.

**Methodology:** Jiaqi Wang, Tengchao Yang.

**Project administration:** Lijun Wei.

**Supervision:** Lijun Wei, Lu Shen.

**Visualization:** Changming Xu, Yun Tian.

**Writing – original draft:** Lijun Wei, Changming Xu, Tengchao Yang, Yun Tian, Lu Shen.
